# Cases of Successful Surgical Operations

**Published:** 1831

**Authors:** 


XXXIX.

CASES OF SUCCESSFUL SURGICAL OPERATIONS. By Dr. C. BRYCE.

DR. B. ACCOMPANIED Lord Cochrane to

Greece. At first opportunities of practice were promising, but subsequent events diminished those opportunities considerably. The following cases which occurred are of sufficient interest and importance for Dr. B. to narrate and for us to report upon.

Case 1. *Amputation at the Shoulder-Joint, with a portion of the Clavicle and Scapula.*

"Camp of the Phalerus. 4th May, 1827. Soldier, æt. 19, spare habit.

Half an hour before his presentation, a cannon-ball had passed across the breast; struck the left shoulder, removing two inches of the humeral extremity of clavicle, the anterior half of the head of humerus, the acromion process, and part of the spine of scapula. The integuments around the joint were extensively destroyed; but the parts beneath the clavicle, and the muscles forming the arm-pit were sound, or simply bruised. The hæmorrhagy was trifling, and the patient, though depressed, had recovered from the first shock of the injury, and cheerfully disposed himself to operation. After cleansing the wound of splinters and coagulated blood, so as to ascertain the full extent of the injury, it was determined upon, to remove with the saw, the fractured extremities of the clavicle and scapula: to turn out the shoulder-joint; and, by forming a flap from the axilla and inferior surface of the arm, bring it to correspond with the integuments above, so as to cover the cavity. Any apprehension of bleeding from the axillary artery was quieted, by ascertaining the ready command an assistant had over the subclavian. Hey's saw was conveniently applied, to fulfil our first intention: depressing the elbow towards the side, a Lisfranc knife was introduced betwixt the glenoid cavity and fragment of head of humerus, and carried close to the bone, near an inch downwards, and then directed outwards to form a flap. The axillary artery was secured with little loss of blood; and the operation was completed by removing the lacerated edges of the upper portion of the wound, and adapting these in some degree to the form of the flap. No stitches were necessary, as with a little management straps retained the integuments in suitable juxtaposition. The usual dressings and bandages were applied. The lad bore the operation well. No bad symptom had supervened on the third day, when the outer dressings were removed, and the wound not appearing irritated from tension, the plaster was not touched. On the fifth day the dressings were entirely changed, little pus had formed, and the pain and swelling of the parts were inconsiderable. The edges of the wound had either united, or the interstices showed healthy granulations. On the following day he was removed to a general hospital, where he recovered steadily, under the treatment of M. Treiber, the surgeon there."

In the second case related by Dr. Bryce the head and neck of the humerus were severely injured by musket-shot, whilst the vessels and soft parts remained comparatively untouched. The head and shattered portion of the bone were excised, and all was doing well, when, in the sixth week, the patient was attacked with enteritis from sleeping in the open air, and died of that disease. The state of the limb was not examined after death, but before that event the patient could use his fore-arm and even support the weight of the limb. Dr. Bryce saw this operation performed in Vienna with success. The arm proved so serviceable to the patient that he afterwards acted as waiter in a coffee-house.

Case 3. *Successful Amputation at the Hip-joint.*

Piræus Bay. May 6, 1827. A soldier, æt. 23, was wounded by a 6lb. shot at the disastrous battle of Athens. The integuments and muscles of the hip and thigh were very extensively torn and removed. The trochanter, neck, and 4 inches of the femur were shattered, the femoral vessels were untouched, and the mass of flesh on the inside was uninjured. The bleeding was inconsiderable, the depression not extreme. The patient was removed to hospital, and it was determined in consultation to remove the limb at the hip-joint, which was done in the following manner.

"The plan of proceeding was readily determined on, and executed without difficulty, in the following manner. Firm pressure being made by the cross-piece of the screw of a tourniquet and a pad on the external iliac, immediately above Poupart's ligament, a convex incision was made across the highest part of the thigh and hip, passing from the inside of the sulcus of the blood-vessels, to an inch and a half behind the trochanter, including in this convexity and extent the torn superior circumference of the wound, and exposing the capsular ligament of the joint.

The femoral artery was now secured above the branching off of the circumflex and profunda. The capsule and round ligament were next divided, the acetabulum exposed, and the head of bone drawn out. The amputating knife was again taken, and, observing the particular shape of the upper incision, a corresponding flap was formed, by a double stroke of the knife, from the inner and under part of the thigh, in which the fractured portions of the bone, and the contused and lacerated soft parts were included. The arteries were now secured, and the wound cleared of blood. Notwithstanding frequent ablutions of the wound with cold water there existed a troublesome oozing of blood, without our being able to detect its sources, by which, and the fatigue of the operation, the patient became exhausted.* Wine and assuring language restored him somewhat. It was evidently dangerous to dress the wound immediately: and leaving therefore its surface uncovered, exposed to the air, (a method frequently had recourse to in other similar cases,) we proceeded to another amputation. By this management the wound became so dry, after a few minutes, as to allow the operation to be satisfactorily finished. The flap covered very well the face of the wound, and was easily retained in proper contact by strips of adhesive plaster. The common dressings were applied, and a double-headed roller was carefully adapted to the peculiar form of the hip. An anodyne was exhibited, and six hours after the operation he was composed, and had slept; no pain nor bleeding from the wound."

No bad consequences followed, and in six weeks the patient was completely cured. The superior success of operations in Greece is attributed to the abstemious diet and simple manners of the natives.

* "Although allowing for this casualty, there was not greater hæmorrhagy than in ordinary amputation of the leg."

